# Effects of Black Soldier Fly Larval (*Hermetia illucens*) Replacement Diet on Hybrid Grouper (*Epinephelus lanceolatus* ♂ × *E. fuscoguttatus* ♀) Through a Multipronged Nutrigenomic Analysis

**DOI:** 10.1155/anu/5795871

**Published:** 2026-07-15

**Authors:** Yan Chen, Bing Chen, Junru Hu, Junming Cao, Minyi Zhong, Hai Huang, Jiun-Yan Loh

**Affiliations:** ^1^ Yazhou Bay Innovation Institute, Hainan Tropical Ocean University, Sanya, 572024, China, qzu.edu.cn; ^2^ College of Fisheries and Life Science, Hainan Tropical Ocean University, Sanya, 572000, China, qzu.edu.cn; ^3^ Hainan Key Laboratory for Conservation and Utilization of Tropical Marine Fishery Resources, Sanya, 572022, Hainan Province, China; ^4^ Institute of Animal Science, Guangdong Academy of Agricultural Sciences, Collaborative Innovation Center of Aquatic Sciences, Key Laboratory of Animal Nutrition and Feed Science in South China, Guangdong Provincial Key Laboratory of Animal Breeding and Nutrition, Ministry of Agriculture and Rural Affairs, Guangzhou, 510640, China, agri.gov.cn; ^5^ Tropical Futures Institute, James Cook University (Singapore Campus), 149 Sims Drive, Singapore, 387380, Singapore

**Keywords:** Black soldier fly larvae, fishmeal, hybrid grouper, physiological response, sustainability, transcriptomic response

## Abstract

This study investigated the effects of replacing fishmeal (FM) protein with black soldier fly larvae (BSFL) in hybrid grouper (*Epinephelus lanceolatus* ♂ × *E. fuscoguttatus* ♀) by integrating growth performance, body composition, serum biochemistry, digestive enzyme activity, histopathology, and transcriptomic analysis. A total of 720 fish (average body weight [ABW]: 56.51 g ± 0.07) were randomly allotted to six isonitrogenous and isocaloric diets (0%, 10%, 20%, 30%, 40%, and 50% FM protein replaced by BSFL), with three replicate tanks per treatment and 40 fish per 500 L tank. After the 56‐day feeding trial, survival remained above 95% in all groups (*p* > 0.05). The highest final weight and growth indices were observed in the BSFL20 group, whereas quadratic regression based on body weight gain (BWG) and specific growth rate (SGR) indicated lower mathematical optima of ~8% BSFL replacement. Feed utilization was improved at 20%–30% BSFL inclusion (*p* < 0.05), but higher inclusion levels reduced growth and feed efficiency. Muscle lipid content and fillet quality were not markedly impaired by moderate BSFL inclusion. Serum biochemical responses and tissue histology suggested that replacement levels above 20% increased physiological stress, with clear pathological changes in liver and stomach tissues at 40%–50% BSFL. Transcriptomic analysis revealed substantial changes in genes related to cell growth, apoptosis, amino acid metabolism, and lipid metabolism, with the strongest liver response observed in the BSFL30 group. Overall, the data support moderate BSFL inclusion as a feasible strategy for partially replacing FM in hybrid grouper, while indicating that excessive replacement compromises physiological stability.

**Summary**

• The best observed growth and feed efficiency among the tested diets occurred at 20% black soldier fly larvae (BSFL) inclusion in the hybrid grouper.
• BSFL levels (>20%) reduced growth and altered liver and stomach histology.
• Transcriptomic analysis revealed metabolic differentially expressed genes (DEGs) at a 30% BSFL replacement.
• BSFL inclusion impacts serum biochemistry and digestive enzyme activity.

## 1. Introduction

Fishmeal (FM) and fish oil have long served as traditional high‐quality protein and lipid sources in aquafeeds [1]. They play a pivotal role in aquaculture, particularly in the production of carnivorous species such as hybrid groupers (*Epinephelus lanceolatus* ♂ × *E. fuscoguttatus* ♀). However, the increasing scarcity of FM and fish oil, driven by the overexploitation of fisheries and rising global demand for seafood, has become a major challenge for the industry [2]. By 2030, an additional 23 million metric tons of marine resources will be required to maintain the current average seafood consumption, underscoring the urgent need for sustainable alternatives [2].

To address the expansion of aquaculture and the shortage of FM, researchers have increasingly explored cost‐effective, high‐quality insect and plant proteins as alternative feed ingredients [1, 3]. This transition is essential for conserving marine resources and promoting the sustainable development of aquaculture [4]. Insect meal production has expanded rapidly in China, North America, Europe, Southeast Asia, and Australia. To date, at least 16 insect species have been evaluated worldwide as potential protein sources for aquatic animals [5, 6], and 16 insect species have been approved for human consumption in Singapore [7]. Available market analyses further suggest strong growth potential for insect‐based ingredients, although specific estimates vary among reports [8, 9]. The edible insect market is projected to reach USD 9.6 billion by 2030, with demand rising to ~3.14 million tons during 2022–2030 [10, 11]. Elleby et al. [12] also estimated that the global production of insect‐based protein powders may exceed 23 million tons by 2030. More specifically, the black soldier fly (BSF) sector is expected to expand rapidly, with the market volume reaching 8.2 million tons by 2033 [13]. In recent decades, China has also developed large‐scale BSF larvae (BSFL) production for use as a protein source and bioenergy substrate in animals such as eels and frogs [14, 15].

Among insect‐derived proteins, BSFL (*Hermetia illucens*) have attracted particular attention because they are rich in antimicrobial peptides (AMPs), which may enhance immunity and support gut microbiota health [16–18]. BSFL are considered a high‐value feed ingredient, with protein levels ranging from 35% to 63% (w/w) and an amino acid profile superior to soybean meal in poultry nutrition [19]. BSFL also contain substantial lipid levels (15%–49%), which can be extracted for biodiesel production, while the defatted meal can be used in the feed industry [20]. Despite these advantages, the use of BSFL as a substitute for FM in aquafeeds has not always produced the expected results [21]. Depending on the fish species, the acceptable level of FM replacement ranges widely from 6% to 100% [22, 23]. Previous studies have shown that increasing BSFL inclusion can impair growth performance in hybrid grouper (*Epinephelus fuscoguttatus* ♀ × *Epinephelus lanceolatus* ♂) [24, 25], rainbow trout, channel catfish (*Ictalurus punctatus*), and turbot (*Psetta maxima*) [26–28]. In contrast, Liu et al. [29] reported positive economic returns when 10% of FM was replaced with BSFL in Asian seabass (*Lates calcarifer*), while lysine and methionine supplementation in Atlantic salmon (*Salmo salar*) diets allowed BSFL inclusion levels of up to 25% [30].

Hybrid grouper is an economically important species in China because of its rapid growth, disease resistance, high market value, and adaptability to both land‐based and marine farming systems. As a carnivorous fish, it requires high dietary protein levels and has traditionally relied heavily on FM, which increases feed costs and constrains sustainability. Although previous studies have examined its protein, lipid, and fatty acid requirements, the feasibility of replacing FM with BSFL and the biologically acceptable inclusion range remain unclear. In our previous work, partial FM replacement with BSFL improved some digestive responses but caused intestinal damage at higher inclusion levels. Therefore, the present study aimed to identify a practical inclusion range for BSFL in hybrid grouper diets. Compared with previous studies focused on single endpoints, this work integrates growth performance, serum biochemistry, histopathology, digestive enzyme activity, and transcriptomic responses to provide a more comprehensive evaluation of BSFL as a dietary protein source for hybrid groupers.

## 2. Materials and Methods

### 2.1. Diet Formulation and Preparation

Six practical diets were formulated to be isonitrogenous (xx%) and isoenergetic (xx%) using FM (Guangzhou Fishtech Biotechnology Co., Ltd., China) and BSFL (Guangzhou Fishtech Biotechnology Co., Ltd., China) as the main protein sources. Soybean oil and fish oil were used as lipid sources, whereas microcrystalline cellulose and pregelatinized starch served as carbohydrate components. FM (crude protein, 57%) was progressively replaced by BSFL (crude protein, 35%; crude lipid, 32%; crude ash, 14.8%) at 10%, 20%, 30%, 40%, and 50% of FM protein while maintaining comparable dietary protein and energy levels. The experimental diets were designated as CK, BSFL10, BSFL20, BSFL30, BSFL40, and BSFL50 (Table 1). No crystalline amino acids, such as lysine or methionine, were supplemented. Table 1 presents the measured proximate indices available for direct comparison.

**Table 1:** Ingredients and proximate compositions of the experimental diets (as dry‐matter basis %).

Ingredients (g 100 g^−1^)	Diets					
	CK	BSFL10	BSFL 20	BSFL30	BSFL40	BSFL50
Fishmeal^a^	40.0	36.0	32.0	28.0	24.0	20.0
BSFL^b^	—	7.43	15.0	22.0	29.71	37.1
Soy protein concentrate (65%)	15.0	15.0	15.0	15.0	15.0	15.0
Casein	5.0	5.0	5.0	5.0	5.0	5.0
Shrimp meal	2.5	2.5	2.5	2.5	2.5	2.5
Wheat flour	16.4	15.27	13.9	12.9	11.29	9.40
Binding agents	3.0	3.0	3.0	3.0	3.0	3.0
Microcrystalline cellulose	2.0	2.0	2.0	2.0	2.0	2.0
Soybean oil	1.0	1.0	1.0	1.0	1.0	1.0
Fish oil	10.1	7.8	5.6	3.6	1.5	0
Squid visceral ointment	1.5	1.5	1.5	1.5	1.5	1.5
Vitamin premix^c^	1.0	1.0	1.00	1.0	1.0	1.0
Mineral premix^d^	1.0	1.0	1.0	1.0	1.0	1.0
Choline chloride	0.5	0.5	0.5	0.5	0.5	0.5
Monocalcium phosphate	1.0	1.0	1.0	1.0	1.0	1.0
Proximate composition						
Crude protein	36.72	36.45	37.5	37.78	37.5	37.75
Crude lipid	14.96	14.67	14.10	14.88	14.27	14.72
Gross energy (kJ g^−1^)	17.21	17.27	17.56	17.34	17.45	17.17
Moisture	8.54	8.23	7.88	7.68	7.40	7.0
Ash	12.10	11.81	11.52	11.20	10.92	10.61

^a^Fish meal: crude protein 57.0%, crude lipid 6.1%, crude fiber 8.4%, crude ash 16.06%, Lys 3.19%, Arg 2.89%, His 2.16%, Asp 6.28%, Glu 9.05%, Gly 4.18%, Ala 4.29%, Val 2.41%, Leu 5.21%, Ile 2.40%, Phe 2.95%, Pro 2.37%, Trp 0.78%, Tyr 1.06%, Ser 2.66%, Met 1.62%, and Thr 3.09%.

^b^Black soldier fly larvae (BSFL): crude protein 35.0%, crude lipid 32%, crude ash 14.8%, Lys 1.76%, Arg 2.31%, His 0.97%, Asp 3.75%, Glu 5.65%, Gly 3.18%, Ala 3.29%, Val 2.41%, Leu 3.21%, Ile 1.64%, Phe 1.65%, Pro 2.37%, Trp 0.78%, Tyr 2.06%, Ser 1.66%, Met 0.56%, and Thr 1.49%.

^c^Vitamin mixture (mg kg^−1^ diet): retinol acetate, 38.0; cholecalciferol, 13.2; α‐tocopherol, 210.0; thiamin, 115.0; riboflavin, 380.0; pyridoxine, 88.0; pantothenic acid, 368.0; niacin acid, 1030.0 biotin, 10.0; folic acid, 20.0; vitamin B12, 1.3; and inositol, 4000.0; ascorbic acid, 500.0.

^d^Mineral mixture (mg kg^−1^ diet): MgSO_4_ · 7H_2_O, 3568.0; NaH_2_PO_4_ · 2H_2_O, 25568.0; KCl, 3020.5; KAI (SO_4_)_2_, 8.3; CoCl_2_, 28.0; ZnSO_4_ · 7H_2_O, 353.0; Ca‐lactate, 15968.0; CuSO_4_ · 5H_2_O, 9.0; KI, 7.0; MnSO_4_ · 4H_2_O, 63.1; Na_2_SeO_3_, 1.5; C_6_H_5_O_7_Fe·5H_2_O, 1533.0; NaCl, 100.0; and NaF, 4.0.

Before extrusion, all ingredients were sieved through a 250 μm mesh, weighed, and mixed thoroughly with distilled water and fish oil at a volume‐to‐weight ratio of 30% to obtain a homogeneous dough. The dough was then extruded into 3 mm pellets using a twin‐screw extruder (F‐26 II, South China University of Technology, China). The pellets were dried in an oven at 60°C and stored at −20°C until use.

### 2.2. Animal Ethics Approval

All animal procedures were approved by the Institutional Animal Care and Utilization Committee of Hainan Tropical Ocean University and the Key Laboratory of Tropical Marine Fishery Resources Protection and Utilization of Hainan Province (Approval Number 20191134A1).

### 2.3. Feeding Trial

#### 2.3.1. Experimental Design

Uniform‐sized hybrid groupers from the same batch were obtained from a commercial hatchery in Hainan, China, and transported to the indoor laboratory of the Key Laboratory of Tropical Marine Fishery Resources Conservation and Utilization of Hainan Province at Hainan Tropical Ocean University (Sanya, Hainan, China). After a 24 h fasting period, 720 fish with an average body weight (ABW) of 56.51 ± 0.07 g were randomly distributed into 18 tanks (500 L per tank; 40 fish per tank) connected to a 10 m^3^ indoor recirculating seawater system (Recycling Water Aquaculture System Co., Ltd., Qingdao, China). Each diet was assigned to three replicate tanks. Before the formal feeding trial, fish were acclimated for 2 weeks and fed a commercial diet (Guangdong Haid Group Co., Ltd., China; crude protein, 40%; crude ash, 16%; crude lipid, 14%; crude fiber, 4%; and total phosphorus, 1.2%) to apparent satiation twice daily at 08:00 and 16:00.

During the experiment, feed intake and mortality were recorded daily. Water quality was monitored daily using a portable multiparameter meter (HM‐B200, Shandong Hengmei Electronic Technology Co., Ltd., China) and remained within acceptable ranges: temperature, 30.15 ± 0.5°C; dissolved oxygen, 7.20 ± 0.3 mg L^−1^; salinity, 29.75 ± 0.5‰; pH, 7.15 ± 0.24; and total ammonia nitrogen, 0.3 ± 0.2 mg L^−1^.

#### 2.3.2. Sample Collection and Growth Performance Determination

At the beginning of the experiment, 20 fish (ABW, 56.55 ± 0.07 g) were sampled, euthanized according to approved animal ethics procedures, homogenized, freeze‐dried, ground, and stored at −20°C for the analysis of initial body composition. At the end of the 8‐week feeding trial, fish were anesthetized with 50 mg L^−1^ MS‐222 (tricaine methanesulfonate; Sigma, USA) before growth measurements and tissue sampling. Twenty fish from each tank were weighed to determine growth‐performance indices, including body weight gain (BWG), weight gain rate (WGR), specific growth rate (SGR), protein efficiency ratio (PER), and feed conversion ratio (FCR). Among these fish, 10 per tank were used for whole‐body composition analysis, and the remaining 10 per tank were dissected for the determination of the hepatosomatic index (HSI), intraperitoneal fat ratio (IPR), and digestive enzyme activities.

For biochemical and histological analyses, an additional 10 fish were randomly sampled from each tank for serum biochemistry and histopathological examination. For transcriptome sequencing, five fish from each tank were randomly selected and anesthetized before tissue collection. This sampling sequence was used to separate growth evaluation from subsequent biochemical, histological, and molecular analyses.

Feed intake was calculated after the removal of residual feed from each feeding event. The following equations were used

Average Body Weight Gain BWG, g=Total Final Weight− Total Initial Weight/ Number of Fish;

Weight Gain Rate WGR,%=100×Final Weight− Initial Weight/ Initial Weight;

Specific Growth Rate SGR,%d−1=100×ln Final Weight–ln Initial Weight/ Total Feeding Days;

FCR= Weight of Diet g/ Weight of Fish Gained g;

Survival Rate %=100×Initial Population – Number of Dead Fish/ Initial Population;

Daily Feed Intake DFI,%d−1=100× Dry Feed Intake/Initial Feed Weight+ Final Weight/2× Duration of Experiment d;

PER= Wet Weight Gain/Protein Intake;

Condition Factor CF, g cm−3=100×Final Weight/Average Body Length of Fish cm3;

HSI %=100×Wet Weight of the Liver/Final Weight of the Total Body Weight;

IPR %=100×Intraperitoneal Fat Weight/Final Weight of the Total Body Weight.

#### 2.3.3. Biochemical Analysis

The nutrient composition of fish samples, including dry matter, crude ash, crude protein, crude lipid, and moisture, was determined according to AOAC methods (1995) (crude protein: AOAC 968.06; crude lipid: AOAC 963.15; moisture: AOAC 930.15 and 950.46; and ash: AOAC 942.05). The gross energy of the diets was measured using an adiabatic calorimeter (C2000, IKA‐Werke GmbH & Co., Staufen, Germany). Serum biochemical indices, including aspartate aminotransferase (AST), alkaline phosphatase (ALP), alanine aminotransferase (ALT), gamma–glutamyl transferase (GGT), uric acid (UA), total bile acids (TBA), and blood urea nitrogen (BUN), were analyzed using an automatic biochemical analyzer (Hitachi 7020, Hitachi Scientific Systems, Ibaraki, Japan).

#### 2.3.4. Histopathological Analysis of Fish Tissues

At the end of the feeding trial, liver samples were collected from the central parenchymal region of the liver lobe (avoiding large blood vessels and portal tracts), and stomach samples were excised from the middle segment of the gastric fundus. All fresh tissues were fixed in 4% paraformaldehyde. Subsequent dehydration, paraffin embedding, sectioning, and hematoxylin–eosin (H&E) staining were performed following standard histological protocols described by Chen et al. [31] and Liu et al. [32].

#### 2.3.5. *D*igestive Enzyme Activities in Fish Hepatopancreas and Intestines

After the 56‐day feeding trial, 1 g of intestinal or liver tissue was collected from each fish and homogenized in 9 mL of normal saline (*n* = 10) on ice. The homogenates were centrifuged at 3500 r/min for 10 min, and the supernatants were collected and stored at −80°C until further analysis. The activities of pepsin, amylase, lipase, and trypsin were determined using commercial assay kits (Nanjing Jiancheng Bioengineering Institute, China) according to the manufacturer’s instructions. Enzyme activities were expressed relative to the tissue weight (g).

#### 2.3.6. Transcriptome Sequencing

Based on the physiological response results, five fish from each tank in the CK, BSFL10, BSFL30, and BSFL50 groups were selected for transcriptome sequencing of the liver and muscle tissues. Tissue samples were pooled to increase the RNA yield and reduce the influence of individual variation, thereby improving data quality.

For library preparation, 1 μg of total RNA from each liver and muscle sample was used to construct sequencing libraries with the NEBNext Ultra RNA Library Prep Kit from Illumina (NEB, USA) according to the manufacturer’s instructions.

Gene expression levels were quantified using HTSeq based on the number of reads mapped to each gene and were normalized as fragments per kilobase of transcript per million mapped reads (FPKM), as described by Mortazavi et al. [33] and shown in the following formula:

FPKM=106CNL103,

FPKM: The expression level of gene A;

C: The number of fragments uniquely aligned to gene A;

N: The total number of fragments aligned to all genes;

L: The number of bases in gene A.

The FPKM method has been demonstrated to be effective in eliminating the influence of varying gene lengths and sequence differences on the quantification of gene expression.

Differentially expressed genes (DEGs) were identified using DEGSeq. Genes with a false discovery rate (FDR) <0.01 and an absolute |log_2_ fold change| > 1 were considered significantly differentially expressed. Gene Ontology (GO) enrichment analysis was performed using the GO database, and Kyoto Encyclopedia of Genes and Genomes (KEGG) pathway enrichment was analyzed using KOBAS.

#### 2.3.7. Quantitative Real‐Time PCR (qRT‐PCR) Validation of Representative DEGs

Representative DEGs related to cell growth/apoptosis, amino acid metabolism, and lipid metabolism in liver tissue were selected for qRT‐PCR validation. Total RNA was extracted from the liver tissue using a TRIzol‐based method with DNase treatment according to Abcam’s protocol for RNA isolation and reverse transcription from tissue samples. RNA concentration and purity were determined spectrophotometrically, and RNA integrity was assessed by agarose gel electrophoresis. First‐strand complementary DNA (cDNA) was synthesized from 200 to 500 ng of total RNA using the Hi‐Fi cDNA Synthesis Kit (ab185916, Abcam) according to the manufacturer’s instructions. Quantitative PCR was performed in 20 μL reaction mixtures containing 10 μL of 2 × SYBR Green qPCR master mix, 1 μL each of forward and reverse primers, 1–2 μL of the cDNA template, and nuclease‐free water. The amplification program consisted of an initial denaturation at 95°C for 3–10 min, followed by 40 cycles of 95°C for 30 s, 60°C for 30 s, and 72°C for 30 s. Melting‐curve analysis was performed to confirm amplification specificity. Relative gene expression levels were calculated using the 2^−ΔΔCt^ method.

### 2.4. Statistical Analysis

All data were first tested for normality and homogeneity of variance. Differences among treatment means were then analyzed by one‐way analysis of variance (ANOVA) using SPSS version 19.0 (SPSS Inc., Chicago, IL, USA), with statistical significance set at *p*  < 0.05. When a significant overall effect was detected, post hoc comparisons were conducted, as described in the corresponding table footnotes. To estimate the dietary replacement level associated with optimal growth, quadratic regression models were fitted separately for BWG and SGR using the BSFL replacement level as the independent variable. Only quadratic regression was used to estimate the optimal replacement level to avoid conflicting interpretations among the different models.

## 3. Results

### 3.1. Growth Performance and Biometric Parameters

Table 2 summarizes the effects of graded BSFL inclusion on the growth performance, survival, and biometric indices of hybrid groupers. Survival remained above 95% in all treatments and did not differ significantly among groups. The highest final weight, BWG, WGR, and SGR were observed in fish fed the BSFL20 diet, whereas the lowest values were recorded in the BSFL50 group, indicating that moderate BSFL inclusion supported growth, whereas excessive replacement impaired performance.

**Table 2:** Growth and biometry of hybrid grouper‐fed diets containing various concentrations of FM replacement with BSFL.

Performances	Diets							
	CK	BSFL 10	BSFL 20	BSFL 30	BSFL 40	BSFL 50	SEM	*p*
*W*_i_ (g)	56.50 ± 0.00	56.47 ± 0.04	56.53 ± 0.09	56.53 ± 0.05	56.49 ± 0.19	56.58 ± 0.06	0.022	0.59
*W*_f_ (g)	108.70 ± 0.24^a^	107.19 ± 0.25^b^	109.32 ± 0.27^a^	95.36 ± 0.04^c^	80.41 ± 0.29^d^	76.55 ± 0.17^e^	0.053	<0.001
BWG (g)	52.21 ± 0.24^a^	50.72 ± 0.26^b^	52.29 ± 0.28^a^	39.13 ± 0.08^c^	23.92 ± 0.16^d^	19.98 ± 0.22^e^	0.051	<0.001
WGR (%)	92.42 ± 0.74^a^	89.83 ± 0.83^a^	91.68 ± 0.95^a^	66.54 ± 1.69^b^	41.07 ± 1.14^c^	34.10 ± 1.56^d^	0.284	<0.001
SGR (% d^−1^)	1.56 ± 0.10^a^	1.53 ± 0.10^b^	1.55 ± 0.14^a^	1.26 ± 0.24^c^	0.84 ± 0.15^d^	0.72 ± 0.03^e^	0.033	<0.001
PER	1.80 ± 0.09^b^	2.40 ± 0.13^a^	2.42 ± 0.10^a^	1.77 ± 0.10^b^	1.11 ± 0.12^c^	1.00 ± 0.11^d^	0.026	<0.001
DFI (% d^−1^)	2.28 ± 0.04^a^	1.95 ± 0.23^b^	1.83 ± 0.28^c^	1.77 ± 0.15^d^	1.69 ± 0.12^e^	1.65 ± 0.14^e^	0.042	<0.001
FCR	1.51 ± 0.08^c^	1.14 ± 0.02^d^	1.10 ± 0.02^d^	1.49 ± 0.07^c^	2.40 ± 0.10^b^	2.65 ± 0.2^a^	0.026	<0.001

*Note:* Values (mean, *n* = 3) within the same row with different letters are significantly different (*p* < 0.05). Absence of letters indicates no significant difference between treatments.

The PER was highest in the BSFL10 and BSFL20 groups and was significantly higher than in the other treatments. Daily feed intake (DFI) decreased progressively with increasing BSFL inclusion and was lowest in the BSFL40 and BSFL50 groups, whereas the highest DFI was recorded in the control group (CK). In contrast, the FCR showed the opposite trend, indicating poorer feed utilization at higher replacement levels.

**Figure 1:**
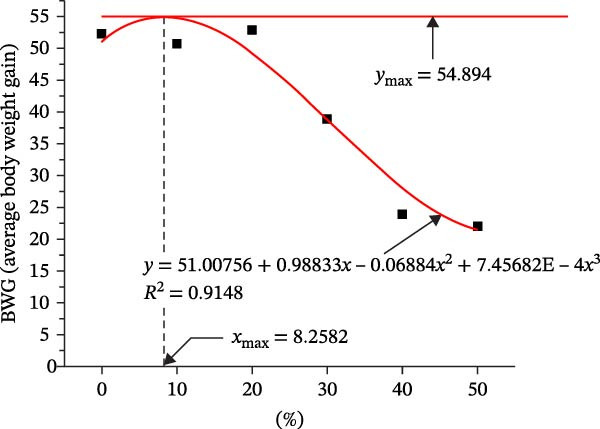
Quadratic regression model was established on body weight gain (*y*‐axis) in response to FM protein replacement level (*x*‐axis) by BSFL.

**Figure 2:**
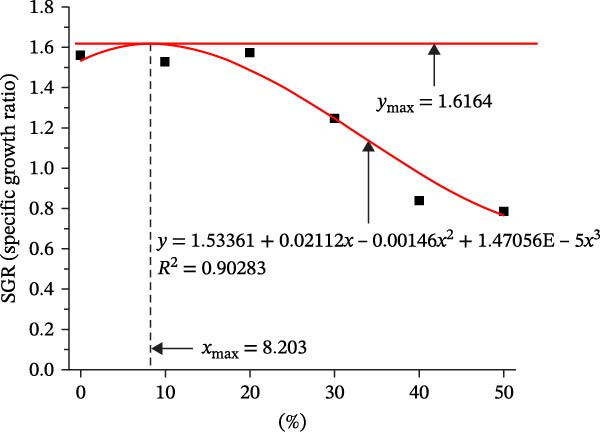
Quadratic regression model was established on specific growth rate (*y*‐axis) in response to FM protein replacement level (*x*‐axis) by BSFL.

Quadratic regression analysis was applied to the two key growth indicators used to estimate the practical replacement optimum. The fitted models indicated estimated optima of 8.26% FM replacement for maximum BWG and 8.20% FM replacement for maximum SGR (Figures 1 and 2). No significant differences in the condition factor (CF) were detected among treatments. The HSI was significantly higher in all BSFL groups than in the CK group (*p* < 0.05), whereas the IPR was highest in the CK group, followed by BSFL20 and BSFL50.

### 3.2. Nutritional Composition of Muscle and Whole Body

Table 3 presents the proximate composition of muscle and whole body in hybrid grouper‐fed diets with graded FM replacement by BSFL. Muscle crude protein content was highest in the CK and BSFL10 groups, followed by BSFL20 (*p* < 0.05), whereas fish fed BSFL30 and BSFL40 showed the lowest values. Significant differences in muscle ash content were detected between BSFL10 and BSFL30, as well as among CK, BSFL20, BSFL40, and BSFL50 (*p* < 0.05). The whole‐body moisture content was highest in the BSFL40 and BSFL50 groups, whereas the lowest value was observed in BSFL10, which did not differ significantly from CK, BSFL20, or BSFL30. Whole‐body crude protein content was the highest in fish fed the CK diet (15.97%) and differed significantly from that in all BSFL‐fed groups. Whole‐body lipid content in the BSFL50 group differed significantly from that in the CK, BSFL20, and BSFL40 groups (*p* < 0.05). In addition, whole‐body ash content in fish fed BSFL20, BSFL30, and BSFL40 differed significantly from that in the CK and BSFL50 groups (*p* < 0.05).

**Table 3:** Proximate composition of muscle and whole body of hybrid grouper‐fed diets containing various concentrations of FM replaced with BSFL.

Nutrients	Diets							
	CK	BSFL 10	BSFL 20	BSFL 30	BSFL 40	BSFL 50	SEM	*p*
Proximate composition of muscle (% WW)								
Moisture	69.75 ± 0.14^c^	69.66 ± 0.23^c^	70.36 ± 0.18^b^	70.34 ± 0.21^b^	70.52 ± 0.46^b^	71.61 ± 0.85^a^	0.100	0.003
Protein	17.08 ± 0.22^a^	17.01 ± 0.14^a^	16.37 ± 0.29^b^	14.46 ± 0.18^d^	14.94 ± 0.18^cd^	15.03 ± 0.04^c^	0.045	<0.001
Lipid	4.07 ± 0.09^a^	4.00 ± 0.05^a^	3.41 ± 0.14^b^	3.92 ± 0.13^a^	4.03 ± 0.27^a^	4.31 ± 0.13^a^	0.036	<0.001
Ash	3.45 ± 0.10^b^	3.91 ± 0.09^a^	3.62 ± 0.05^b^	3.94 ± 0.13^a^	3.47 ± 0.27^b^	3.52 ± 0.13^b^	0.034	<0.001
Whole fish								
Moisture	77.36 ± 0.24^c^	77.03 ± 0.38^c^	77.59 ± 0.36^c^	78.03 ± 0.43^bc^	79.18 ± 0.43^a^	78.68 ± 0.27^ab^	0.072	<0.001
Protein	15.97 ± 0.13^a^	14.11 ± 0.14^b^	13.63 ± 0.26^c^	13.15 ± 0.22^e^	12.47 ± 0.20^d^	13.74 ± 0.12^bc^	0.044	<0.001
Lipid	7.74 ± 0.08^b^	7.17 ± 0.02^cd^	7.68 ± 0.25^b^	6.80 ± 0.18^d^	7.36 ± 0.15^bc^	8.45 ± 0.24^a^	0.040	<0.001
Ash	4.93 ± 0.06^d^	5.45 ± 0.18^bc^	5.88 ± 0.09^a^	5.85 ± 0.05^a^	5.72 ± 0.09^ab^	5.21 ± 0.15^cd^	0.118	<0.001

*Note*: Values (mean, *n* = 3) within the same row with different letters are significantly different (*p* < 0.05). Absence of letters indicates no significant difference between treatments.

Abbreviation: WW, wet weight.

### 3.3. Blood Serum Biochemistry

Table 4 shows the serum biochemical responses of hybrid grouper‐fed diets containing graded levels of BSFL in place of FM. Serum ALT activity was significantly higher in the BSFL10, BSFL20, and BSFL40 groups than in the CK group and the other BSFL groups (*p* < 0.05), whereas AST activity did not differ significantly among treatments. The AST/ALT ratio was also significantly altered among groups and was higher in the BSFL treatment groups than in the CK group (*p* < 0.05).

**Table 4:** Blood serum biochemistry of hybrid grouper‐fed different diets.

Biomarkers	Groups							
	CK	BSFL 10	BSFL 20	BSFL 30	BSFL 40	BSFL 50	SEM	*p*
Liver function								
ALT (U/L)	182.91 ± 5.21^d^	207.66 ± 17.66^b^	190.74 ± 17.82^c^	172.74 ± 4.78^e^	229.47 ± 4.12^a^	173.90 ± 5.17^e^	2.59	<0.001
AST (U/L)	229.81 ± 5.66^a^	202.21 ± 6.15^ab^	162.60 ± 4.95^ab^	85.05 ± 2.67^b^	163.43 ± 2.36^ab^	88.41 ± 2.99^b^	1.04	<0.001
AST/ALT	1.94 ± 0.90^a^	0.95 ± 0.27^b^	0.88 ± 0.32^b^	0.49 ± 0.25^d^	1.21 ± 0.57^ab^	0.68 ± 0.29^c^	0.12	0.012
r‐GT (U/L)	11.99 ± 1.85	6.28 ± 0.29	8.62 ± 1.24	10.63 ± 0.98	18.38 ± 2.86	16.95 ± 2.95	0.46	0.181
ALP (U/L)	339.68 ± 93.49^c^	389.65 ± 12.0^c^	539.24 ± 11.76^c^	597.72 ± 9.67^bc^	1053.49 ± 7.56^ab^	1143.56 ± 7.57^a^	9.24	<0.001
TBA (μmol/L)	21.63 ± 1.98^a^	14.37 ± 3.79^c^	12.90 ± 1.36^c^	17.18 ± 2.61^b^	17.19 ± 0.74^b^	17.23 ± 0.73^b^	0.51	<0.001
Kidney function								
BUN (mg/dL)	20.97 ± 2.58^b^	24.57 ± 5.63^a^	20.95 ± 5.74^b^	24.92 ± 8.37^a^	25.55 ± 4.63^a^	20.24 ± 4.14^b^	1.29	<0.001
UA (μmol/L)	307.34 ± 5.67^e^	393.19 ± 13.94^d^	718.27 ± 6.90^b^	741.37 ± 9.31^a^	419.76 ± 24.57^c^	427.70 ± 22.83^c^	3.71	0.002

*Note:* Values (mean; *n* = 3) within the same row with different letters were significantly different (*p* < 0.05). The absence of letters indicates no significant difference between treatments.

Abbreviations: ALP, alkaline phosphatase; ALT, alanine transaminase; AST, aspartate aminotransferase; BUN, blood urea nitrogen; r‐GT, r‐glutamyl transferase; TBA, total bile acids; UA, uric acid.

GGT activity did not differ significantly among treatments (*p* > 0.05). In contrast, ALP activity increased significantly with increasing BSFL inclusion. Serum TBA levels were higher in the CK group than in all BSFL groups, with the lowest values observed in BSFL10 and BSFL20 (*p* < 0.05). BUN varied among treatments and was higher in BSFL10, BSFL30, and BSFL40 than in CK, BSFL20, and BSFL50 (*p* < 0.05). UA also differed significantly among groups, with the highest values in BSFL20 and BSFL30 and the lowest value in the CK group (*p* < 0.05).

### 3.4. Digestive Enzymatic Activity

Activities of pepsin, amylase, lipase, and trypsin differed significantly among dietary treatments (*p* < 0.05; Table 5). In the liver, fish fed BSFL10 showed significantly higher pepsin and lipase activities than those in the CK, BSFL20, and BSFL30 groups. Amylase activity was the highest in fish fed BSFL20 and was significantly higher than that in BSFL10. Trypsin activity differed significantly in fish fed BSFL40 and BSFL50 compared with the CK and the other BSFL groups (*p* < 0.05).

**Table 5:** Enzyme activities of juvenile hybrid grouper‐fed different diets.

Organs enzymes		Groups							
		CK	BSFL 10	BSFL 20	BSFL 30	BSFL 40	BSFL 50	SEM	*p*
Liver	Pepsin (U/mg protein)	0.19 ± 0.01^c^	0.87 ± 0.01^a^	0.12 ± 0.03^c^	0.33 ± 0.01^b^	0.27 ± 0.16^b^	0.36 ± 0.17^b^	0.023	<0.001
	Amylase (U/mg protein)	0.43 ± 0.03^ab^	0.28 ± 0.12^b^	0.55 ± 0.02^a^	0.46 ± 0.11^ab^	0.42 ± 0.08^ab^	0.51 ± 0.06^ab^	0.019	0.004
	Lipase (U/g)	0.61 ± 0.07^b^	0.99 ± 0.04^a^	0.67 ± 0.13^b^	0.64 ± 0.16^b^	0.75 ± 0.05^ab^	0.70 ± 0.04^ab^	0.022	<0.001
	Trypsin (U/mg protein)	37.26 ± 6.26^b^	52.67 ± 13.38^b^	39.19 ± 5.28^b^	53.73 ± 27.92^b^	165.31 ± 25.33^a^	125.28 ± 12.01^a^	4.09	<0.001
	Pepsin (U/mg protein)	0.61 ± 0.11^a^	0.32 ± 0.02^b^	0.28 ± 0.16^b^	0.11 ± 0.01^c^	0.32 ± 0.04^b^	0.06 ± 0.01^c^	0.019	<0.001
Stomach	Amylase (U/mg protein)	0.05 ± 0.00	0.05 ± 0.00	0.04 ± 0.00	0.04 ± 0.00	0.05 ± 0.00	0.05 ± 0.00	0.00	1.00
	Lipase (U/g)	1.18 ± 0.11^ab^	1.51 ± 0.19^a^	0.73 ± 0.24^b^	1.30 ± 0.14^ab^	1.12 ± 0.22^ab^	1.25 ± 0.18^ab^	0.044	<0.001
	Trypsin (U/mg protein)	95.97 ± 22.42 ^ab^	128.42 ± 18.12^ab^	71.05 ± 20.43^ab^	181.15 ± 17.58^a^	37.52 ± 5.70^b^	105.01 ± 5.21^ab^	3.87	<0.001

*Note:* Values (mean, *n* = 3) within the same row with different letters were significantly different (*p* < 0.05). The absence of letters indicates no significant difference between treatments.

In the stomach, pepsin activity was significantly higher in fish fed the CK diet than in all BSFL‐fed groups (*p* < 0.05). Lipase activity was the highest in BSFL10 and the lowest in BSFL20. Stomach trypsin activity in BSFL30 differed significantly from that in BSFL40 but did not differ from that in the other groups (*p* < 0.05).

### 3.5. Hepatic Histopathology

**Figure 3:**
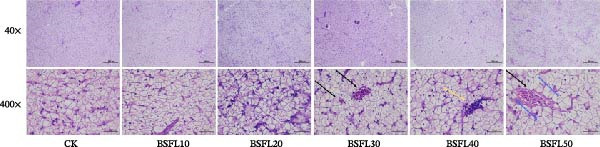
H&E‐stained liver tissue sections of juvenile hybrid grouper‐fed different diets. Upper panels: 40× magnification (scale bar = 500 μm); lower panels: 400× magnification (scale bar = 50 μm). Black arrows indicate mild congestion in hepatic sinusoids and hepatic veins; yellow arrows mark focal infiltration of lymphocytes in partial hepatic lobules; blue arrows show obvious swelling of hepatocytes.

As shown in Figure 3, hepatocytes in the CK, BSFL10, BSFL20, and BSFL30 groups exhibited a well‐organized structure with distinct cell boundaries and clearly visible nuclei. However, when the BSFL replacement level increased to 40% and 50%, there was mild congestion in the hepatic sinusoids and hepatic veins. Occasionally, focal lymphocyte infiltration and significant hepatocyte swelling can be observed in the local liver lobules.

**Figure 4:**
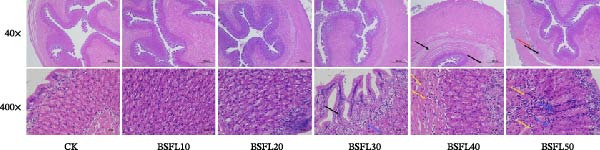
H&E‐stained stomach tissue sections of juvenile hybrid grouper‐fed different diets. Upper panels: 40× magnification (scale bar = 500 μm); lower panels: 400× magnification (scale bar = 50 μm). Black arrows point to sparse mucous glands in the upper lamina propria and dilated blood vessels in the submucosa; blue arrows indicate abundant glands with intensely eosinophilic cytoplasm beneath mucous glands; yellow arrows show scattered eosinophil infiltration in the muscularis mucosae; red arrows mark focal inflammatory cell infiltration within the submucosa.

In the stomach, fish fed the CK, BSFL10, BSFL20, and BSFL30 diets showed a well‐developed gastric mucosa (Figure 4). Epithelial cells were arranged regularly, with no obvious shedding. The glands in the lamina propria were abundant and densely distributed, and no evident inflammatory cell infiltration was observed. In contrast, fish fed BSFL40 and BSFL50 showed a reduced number of mucinous glands in the upper lamina propria, increased eosinophilic glands in the lower lamina propria, and localized lymphocytic infiltration. In addition, a small number of eosinophilic granulocytes were observed in the muscularis mucosa, together with localized dilation of submucosal vessels, indicating localized inflammatory infiltration.

### 3.6. Transcript Counts

Table 6 summarizes the transcriptome sequencing output from muscle, liver, and intestine samples of juvenile hybrid grouper under different dietary treatments. A total of 32.2 GB of clean data was generated, comprising 51.25 million clean reads with an average read length of 150 bp. The number of clean reads varied by less than 10% among groups: CK, 4.27 million (liver) and 4.26 million (muscle); BSFL10, 4.23 and 4.67 million; BSFL30, 4.27 and 4.22 million; and BSFL50, 4.35 and 4.69 million.

**Table 6:** Basic data of RNA‐Seq in muscle and liver of juvenile hybrid grouper‐fed with different diets in comparisons of CK, BSFL 10, BSFL 30, and BSFL 50.

Sample	Muscle					Liver			
Groups	CK	BSFL 10	BSFL 30	BSFL 50		CK	BSFL 10	BSFL 30	BSFL 50
Read length	150	150	150	150		150	150	150	150
Clean reads (×10^7^)	4.26	4.67	4.22	4.68		4.27	4.23	4.27	4.35
Adapter read (×10^5^)	7.57	10.84	10.31	9.16		10.38	13.70	10.71	9.02
GC (%)	52.19	52.62	51.84	52.36		49.72	49.17	48.95	49.08
Cleans Q20 (%)	97.8	97.83	97.78	97.77		97.86	98.03	97.94	97.74
Cleans Q30 (%)	93.81	93.89	93.71	93.72		93.84	94.2	93.97	93.54

**Figure 5:**
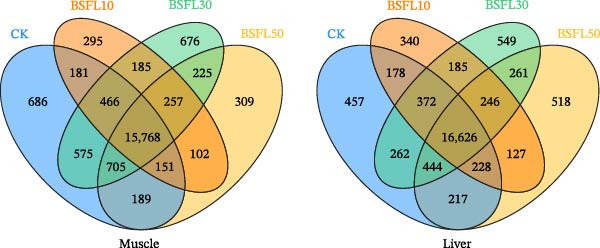
Venn diagram showing the number of transcripts in muscle, liver, and intestine of juvenile hybrid grouper‐fed different diets by comparing two contrasts.

Based on these data, transcriptomic responses were compared among CK, BSFL10, BSFL30, and BSFL50 groups (Figure 5). In total, 100,802, 104,856, and 118,285 shared transcripts were identified in the muscle, liver, and intestine, respectively. In muscle, transcript counts were 18,721, 17,303, 18,857, and 17,706 for CK, BSFL10, BSFL30, and BSFL50, respectively. In pairwise comparisons of CK vs. BSFL10, CK vs. BSFL30, CK vs. BSFL50, BSFL10 vs. BSFL30, BSFL10 vs. BSFL50, and BSFL30 vs. BSFL50, the numbers of shared transcripts were 16,566, 16,676, 16,955, 17,514, 16,813, and 16,278, respectively. In liver, transcript counts were 18,784, 18,302, 18,945, and 18,667 for CK, BSFL10, BSFL30, and BSFL50, respectively, with 17,404, 17,704, 17,515, 17,429, 17,227, and 17,577 shared transcripts across the same comparisons. In the intestine, transcript counts were 20,813, 20,158, 20,743, and 20,984, respectively, with 19,511, 19,832, 19,971, 19,457, 19,600, and 19,914 shared transcripts among pairwise comparisons.

### 3.7. Extensive Transcriptional Reprogramming of DEGs

**Figure 6:**
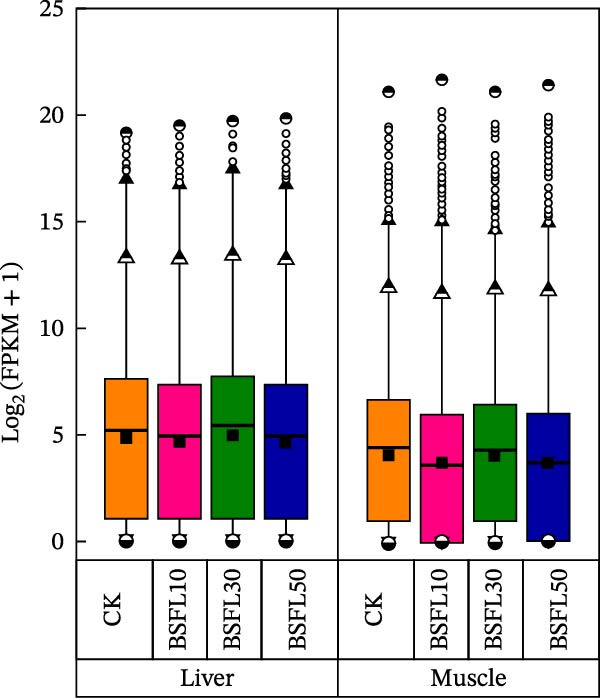
Boxplot analysis of expression levels of DEGs in muscle and liver of the hybrid grouper.

Figure 6 shows boxplots of DEG expression levels in the muscle and liver tissues. In the liver, the median DEG expression level in BSFL30 was significantly higher than that in BSFL10 and BSFL50 (*p* < 0.05), suggesting a stronger transcriptional response at the 30% replacement level. The CK group showed intermediate expression, whereas BSFL10 and BSFL50 showed relatively lower levels. The greater variability in BSFL10 and BSFL50 suggested increased heterogeneity in transcriptional responses. In muscle, lower DEG expression levels were observed in BSFL10 and BSFL50 than in CK and BSFL30, indicating a narrower range of transcriptional changes at these replacement levels.

**Figure 7:**
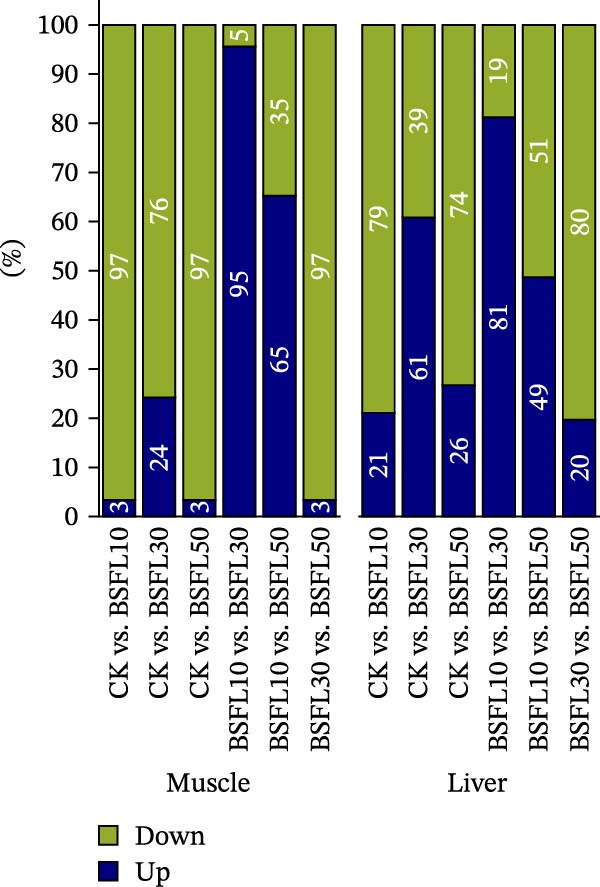
Statistics of the differentially expressed genes (DEGs) in muscle, liver, and intestine of juvenile hybrid grouper‐fed different diets in comparisons of two groups.

Figure 7 summarizes the numbers of upregulated and downregulated DEGs among CK, BSFL10, BSFL30, and BSFL50. In muscle, the numbers of DEGs were 4041 (CK vs. BSFL10), 1387 (CK vs. BSFL30), 3394 (CK vs. BSFL50), 2597 (BSFL10 vs. BSFL30), 486 (BSFL10 vs. BSFL50), and 1180 (BSFL30 vs. BSFL50). Among these comparisons, BSFL30 showed the highest proportion of upregulated DEGs (24%) relative to that of CK, BSFL10, and BSFL50. In the comparison of BSFL30 vs. BSFL50, the proportion of downregulated DEGs was more than 30‐fold higher, suggesting that the BSFL30 diet tended to induce transcriptional activation in the muscle. In the liver, the numbers of DEGs were 1971 (CK vs. BSFL10), 3068 (CK vs. BSFL30), 3062 (CK vs. BSFL50), 3159 (BSFL10 vs. BSFL30), 2105 (BSFL10 vs. BSFL50), and 3484 (BSFL30 vs. BSFL50). A similar pattern was observed in the liver, where BSFL30 showed 61% upregulated DEGs, whereas BSFL10 showed only 21%.

### 3.8. Enrichment of Functional Analysis of DEGs

All transcripts were subjected to functional enrichment and classification analyses using the GO and KEGG pathways. GO classifications for the muscle and liver are shown in Figure 8A,B.

Figure 8GO classification of all DEGs in muscle and liver of hybrid grouper. (A) Enriched GO terms of muscle and (B) Enriched GO terms of liver. BPs: biological processes; CCs: cellular components; MFs: molecular functions.

**Figure:**
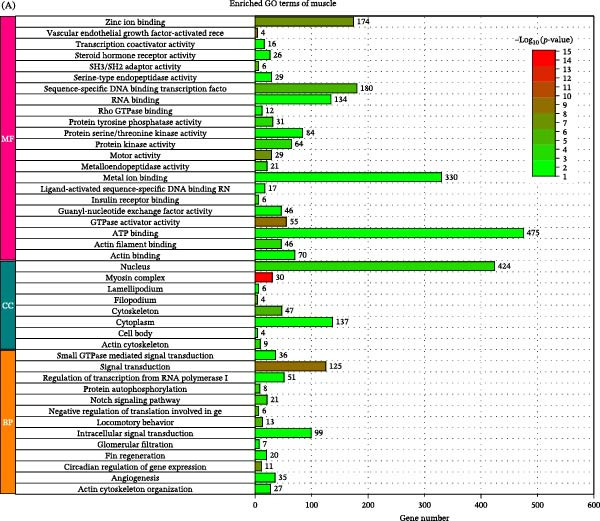

**Figure:**
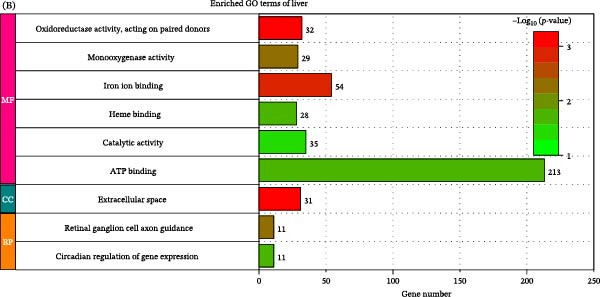

In muscle, 2975 unigenes were annotated in the GO database and assigned to three main categories: molecular function (MF), cellular component (CC), and biological process (BP). The MF category included 22 subcategories, the CC category included 8 subcategories, and the BP category included 13 subcategories. Within MF, the most abundant terms were mainly related to binding and catalytic activity, especially ATP binding, metal ion binding, sequence‐specific DNA‐binding transcription factor activity, RNA binding, and zinc ion binding, which together accounted for most annotated unigenes. Significant differences were observed in zinc–ion binding, SH3/SH2 adaptor activity, sequence‐specific DNA‐binding transcription factor activity, motor activity, and GTPase activator activity.

Within the CC category, the most abundant terms were the nucleus, cytoskeleton, and myosin complex, which together accounted for most annotated unigenes. Significant differences were also detected between these terms. Within the BP category, signal transduction‐related terms were prominent, including small GTPase‐mediated signal transduction, intracellular signal transduction, regulation of transcription from RNA polymerase I, and angiogenesis. Significant differences were also identified in signal transduction, negative regulation of translation involved in gene expression, locomotory behavior, and circadian regulation of gene expression.

In the liver, CCs were classified into six groups, MFs into one major group, and BPs into two categories. Within MF, significant differences were observed in catalytic activity, oxidoreductase activity acting on paired donors, monooxygenase activity, iron‐ion binding, heme binding, and ATP binding. A total of 22 enriched GO terms were identified in BP, with notable differences in retinal ganglion cell axon guidance and circadian regulation of gene expression. In the CC category, the extracellular space was the dominant term and also showed significant variation.

Overall, DEG analysis indicated that most genes were downregulated in both muscle (68%) and liver (57%), suggesting that FM replacement by BSFL substantially altered the cellular composition, metabolic processes, and MFs in both tissues.

### 3.9. KEGG Enrichment Analysis

KEGG enrichment analysis further clarified the functional characteristics of the DEGs (Figure 9A,B). The annotated pathways were assigned to major categories, including cellular processes, environmental information processing, genetic information processing, metabolism, and organismal systems.

Figure 9KEGG classification of all unigenes in muscle and liver of hybrid grouper. (A) KEGG enrichment for muscle and (B) KEGG enrichment for liver.

**Figure:**
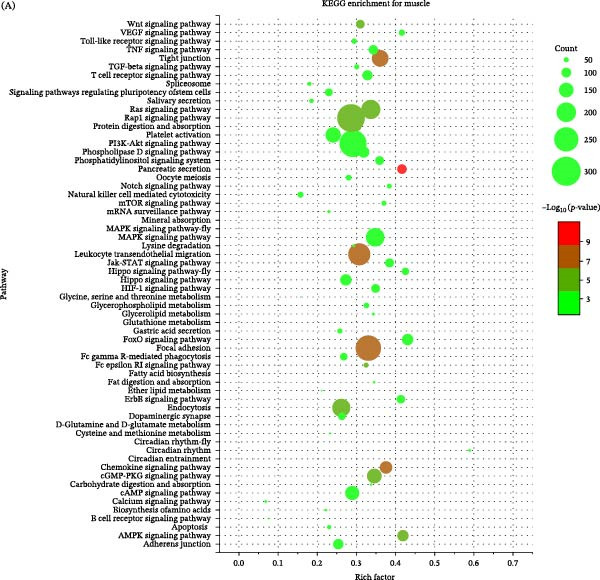

**Figure:**
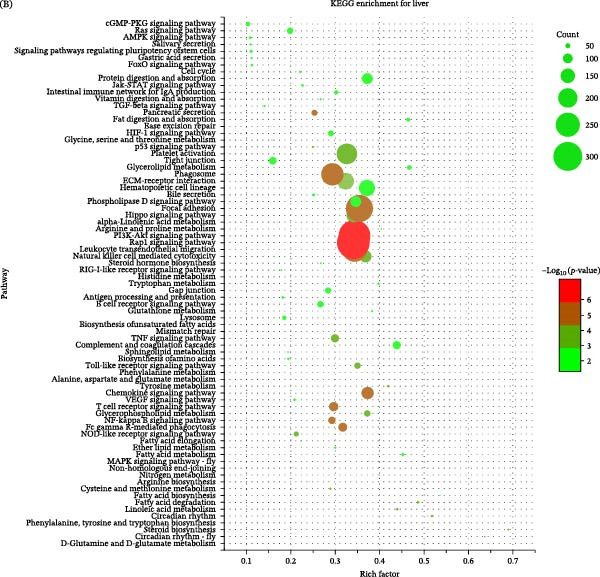

In muscle, environmental information processing accounted for the largest proportion of DEGs (Figure 9A). Among the enriched pathways, Rap1 signaling, phosphatidylinositol 3‐kinase/protein kinase B (PI3K‐Akt) signaling, and focal adhesion contained the largest numbers of DEGs. In addition, Wnt signaling, tight junction, Ras signaling, pancreatic secretion, protein digestion and absorption, leukocyte transendothelial migration, endocytosis, chemokine signaling, cyclic guanosine monophosphate‐protein kinase G (cGMP‐PKG) signaling, and adenosine 5^′^‐monophosphate‐activated protein kinase (AMPK) signaling showed significant enrichment, whereas other pathways contained fewer than 50 DEGs.

In the liver (Figure 9B), organismal systems contributed the highest proportion of DEGs, followed by metabolism. Phagosome, focal adhesion, arginine and proline metabolism, PI3K‐Akt signaling, and Rap1 signaling each contained more than 200 DEGs. Pathways including pancreatic secretion, platelet activation, leukocyte transendothelial migration, chemokine signaling, T‐cell receptor signaling, nuclear factor kappa B (NF‐κB) signaling, and Fc gamma R‐mediated phagocytosis were also significantly enriched, whereas other pathways contained fewer than 50 DEGs.

### 3.10. Heatmap Analysis of DEGs Associated With Cellular Metabolism and Metabolic Processes

The expression patterns of DEGs related to cell growth and apoptosis in the muscle and liver are shown in Figure 10A. In the liver, the expression of several genes, including *igf1*, *ngfb*, *orc2*, *ccnd2a*, *mdm2*, *rela*, *cdkn1a*, *igfbp3*, *mapk8a*, *e2f3*, and *ddb2*, gradually decreased with increasing BSFL replacement. In contrast, *aifm2*, *wee2*, *chek2*, *cdkn1d*, *mdm4*, *casp7*, and *casp9* increased. The BSFL30 group showed marked changes in genes such as *cdkn2c*, *napsa*, *bcl2l1*, *tnfrsfa*, *ccna2*, *ccnb1*, *apaf1*, and *tp53i3*, indicating an active transcriptional response of apoptosis‐related pathways at this replacement level. The expression of *igf3*, *actb1*, *badb*, *trpm7*, *ikbkg*, *cd82b*, and *gsk3ba* remained relatively stable among groups. In muscle, expression levels of *wee2*, *orc2*, *tfr1a*, *ctss2.1*, *tp53i3*, and *ctso* increased with increasing BSFL replacement, whereas *aifm2*, *igf1*, *cdkn2c*, *chek2*, *orc3*, *actb1*, *ddit3*, *gadd45aa*, *zgc:55558*, *gclm*, *ccne1*, and *ddb2* decreased. The BSFL30 group also showed marked shifts in *ccnd2a*, *napsa*, *badb*, *trpm7*, *ikbkg*, *gsk3ba*, *bcl2l1*, *tnfrsfa*, *mdm4*, *rela*, *ccnb1*, *mapk8a*, *casp7*, and *ikbkb*, whereas *prkacba*, *ccnd1*, *ppm1da*, *tgfb1a*, *atm*, *mapk8b*, *cdk2*, and *anapc5* exhibited the highest expression levels among treatments.

Figure 10Differential expression analysis of genes related to (A) cell growth and death in muscle and liver; (B) amino acid metabolism in muscle and liver; and (C) lipid metabolism in muscle and liver.

**Figure:**
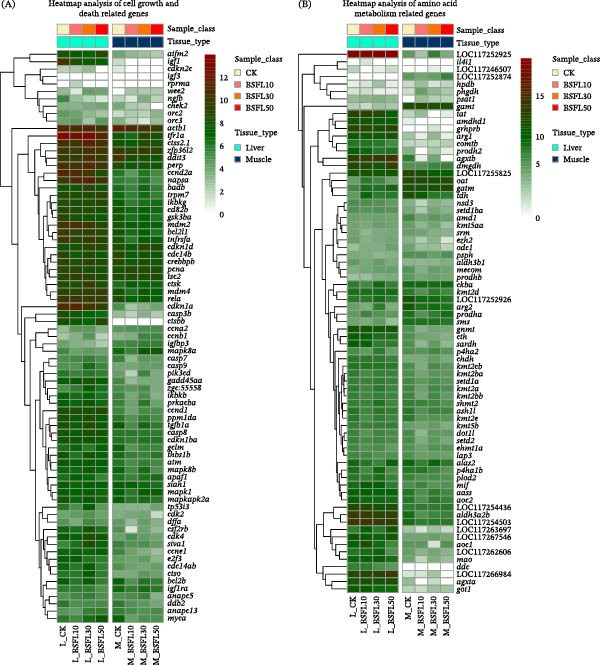

**Figure:**
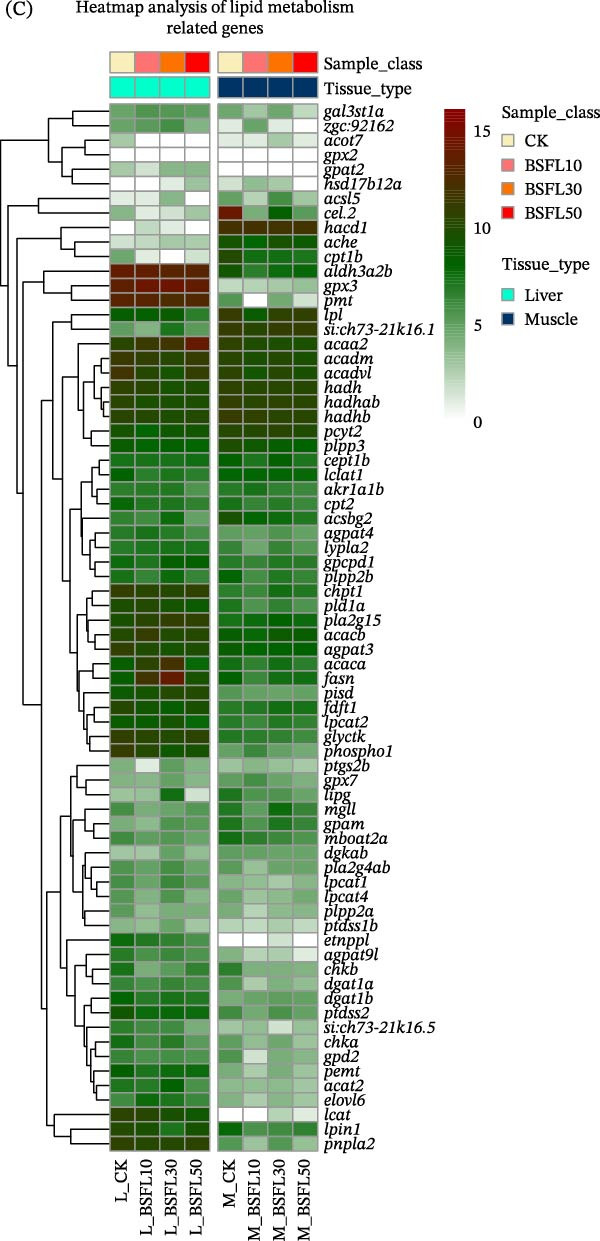

DEGs related to the amino acid metabolism are shown in Figure 10B. In the liver, *agxtb*, *odc1*, *mecom*, and *prodhb* increased with increasing BSFL replacement, whereas *psph*, *cth*, and *sardh* gradually decreased. The BSFL30 group exhibited marked transcriptional responses involving *hpdb*, *gamt*, *oat*, *kmt5aa*, *arg2*, *sms*, *p4ha2*, *kmt2a*, *kmt2bb*, *shmt2*, *dot1l*, *setd2*, *ehmt1a*, *p4ha1b*, and *plod2*. In contrast, the highest expression levels of *il4i1*, *phgdh*, *tdh*, and *prodha* were observed in BSFL10. In muscle, pronounced changes in amino acid metabolism‐related genes were observed in BSFL30, including *LOC117252925*, *LOC117246507*, *arg1*, *prodh2*, *agxtb*, *nsd3*, *setd1ba*, *kmt5aa*, *ezh2*, *kmt2d*, *sardh*, *chdh*, *LOC117262606*, *mao*, *LOC117266984*, *agxta*, *kmt2cb*, *kmt2ba*, *setd1a*, *kmt2a*, *kmt2bb*, *ash1l*, *dot1l*, *setd2*, *ehmt1a*, *aass*, and *aoc2*.

As shown in Figure 10C, several lipid metabolism‐related genes in the liver, including *gpat2*, *hsd17b12a*, *ache*, *acaa2*, *gpcpd1*, *pla2g15*, and *pisd*, were upregulated with increasing BSFL inclusion. In contrast, *acot7*, *cpt1b*, *agpat3*, *glyctk*, *phospho1*, *mboat2a*, *plpp2a*, *si:ch73 -21k16.5*, and *chka* were downregulated. The BSFL30 group showed the highest expression levels of *acsl5*, *si:ch73 -21k16.1*, *lclat1*, *acsbg2*, *plpp2b*, *ptgs2b*, *gpx7*, *lipg*, *dgkab*, *pla2g4ab*, *lpcat1*, *lpcat4*, *ptdss1b*, *zgc:92162*, *lpl*, *acaca*, and *fasn* relative to the other groups.

In muscle, significant changes were observed in *gal3st1a*, *acot7*, *acsl5*, *cel.2*, *pnpla2*, *lcat*, *pemt*, *chka*, *gpd2*, *ptdss2*, *lypla2*, *zgc:92162*, *gpx7*, and *phospho1*, with *lypla2*, *zgc:92162*, *gpx7*, and *phospho1* showing the highest expression levels. By contrast, the expression of *lpin1*, *chkb*, *dgat1a*, *agpat9l*, *lipg*, *pld1a*, and *acsbg2* decreased as the BSFL replacement level increased.

### 3.11. qPCR Validation of Representative DEGs

**Figure 11:**
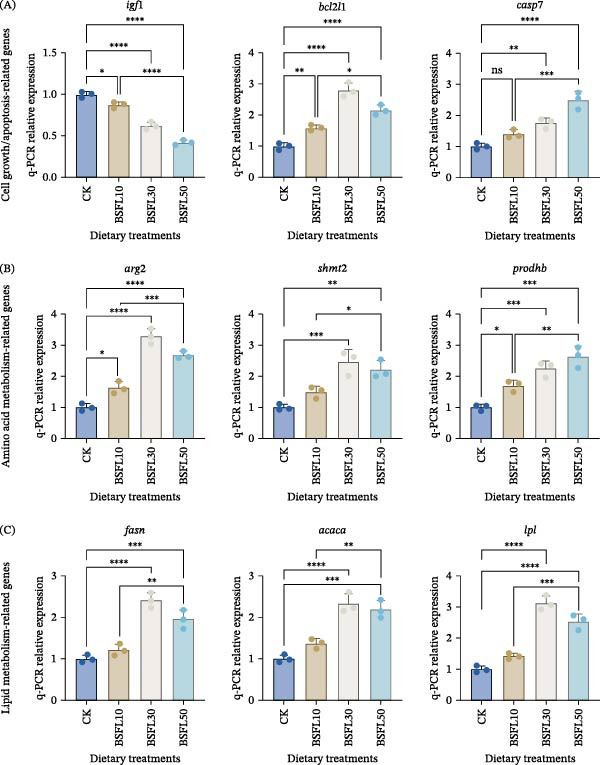
qPCR validation of representative differentially expressed genes (DEGs) in the liver of hybrid grouper‐fed diets with different BSFL replacement levels. (A) Cell growth/apoptosis‐related genes (igf1, bcl2l1, and casp7); (B) amino acid metabolism‐related genes (arg2, shmt2, and prodhb); and (C) lipid metabolism‐related genes (fasn, acaca, and lpl). Data are presented as means ± SD. Statistical differences among dietary treatments are indicated in the figure. Statistical differences among dietary treatments are indicated as  ^∗^ *p*  < 0.05,  ^∗∗^ *p*  < 0.01,  ^∗∗∗^ *p*  < 0.001,  ^∗∗∗∗^ *p*  < 0.0001, and ns (not significant).

Nine representative DEGs involved in cell growth/apoptosis, amino acid metabolism, and lipid metabolism in the liver tissue were selected for qRT‐PCR validation (Figure 11). Overall, the qRT‐PCR results showed expression patterns generally consistent with those obtained by RNA sequencing (RNA‐seq). In the cell growth/apoptosis category, *igf1* showed a decreasing trend with increasing BSFL replacement, whereas *bcl2l1* and *casp7* showed increasing trends. In the amino acid metabolism category, *arg2*, *shmt2*, and *prodhb* were upregulated in the BSFL groups relative to CK, with stronger responses at higher replacement levels. In the lipid metabolism category, *fasn*, *acaca*, and *lpl* showed elevated expression in BSFL30 relative to that in CK and BSFL10. Although the fold‐change magnitudes differed between the two methods for some genes, the overall directions of regulation were consistent, supporting the major pathway‐level interpretations derived from the transcriptomic data.

## 4. Discussion

In the present study, survival remained high across all treatments, whereas growth responses showed a clear nonlinear pattern with increasing BSFL inclusion. The best observed growth performance was recorded in the BSFL20 group, while quadratic regression of BWG and SGR suggested lower mathematical optima of ~8%. Taken together, these findings indicate that low‐to‐moderate BSFL inclusion supports growth in hybrid groupers, whereas replacement levels above 20% begin to impair growth performance and feed utilization. Thus, although the calculated optimum was near 8%, the best observed performance among the tested diets occurred at a 20% replacement.

Previous studies have shown that insect meals are often utilized less efficiently by some cold‐water species, such as rainbow trout and turbot, than by many warm‐water or tropical carnivorous fish. The present results are broadly consistent with this Hybrid grouper tolerated moderate BSFL inclusion without loss of survival and maintained acceptable growth at low‐to‐moderate replacement levels, whereas excessive inclusion impaired the performance. These findings support the view that tropical carnivorous fish may accommodate insect‐derived protein more effectively than some cold‐water species, although species‐specific evaluation remains necessary.

The significant variation in muscle and whole‐body composition observed in this study suggests that the BSFL inclusion level must be optimized to maintain nutritional balance and protein quality. In the absence of supplemental crystalline amino acids, the present results are consistent with previous reports showing that increasing BSFL inclusion may alter nutrient composition and reduce protein digestibility [34]. In addition, serum biochemical responses indicated that dietary BSFL influenced the metabolic status. Fish fed BSFL10, BSFL20, and BSFL40 showed increased ALT activity, suggesting possible hepatic stress. Variations in the AST/ALT ratio, together with increases in ALP and UA at higher inclusion levels, further support the view that excessive BSFL replacement may disturb metabolism and induce physiological stress, in agreement with previous observations in fish fed insect‐based diets [35, 36]. Changes in TBA and BUN also suggest adaptive physiological regulation in response to dietary BSFL. Digestive enzyme activity further supports the view that moderate BSFL inclusion may be better utilized than high inclusion levels. Pepsin and lipase activities were elevated in fish fed BSFL10, which may reflect enhanced digestion of the higher protein and lipid fractions in BSFL‐containing diets, as also reported in *Betta splendens* [37]. Furthermore, the highest amylase activity in BSFL20 may indicate improved digestive adaptation at moderate replacement levels, consistent with findings in European sea bass fed alternative protein sources [38, 39].

Histopathological observations also revealed a clear inclusion‐level effect. No marked liver abnormalities were observed in fish fed the CK, BSFL10, BSFL20, or BSFL30 diets, consistent with previous studies showing that moderate inclusion of BSFL or other alternative protein sources is generally well‐tolerated [37]. In contrast, fish fed BSFL40 and BSFL50 showed clear hepatic deterioration, including vacuolation, lipid accumulation, and nuclear dissolution. These results suggest that high BSFL inclusion adversely affects the liver structure and function. One possible explanation is the elevated chitin content of BSFL, which may reduce nutrient availability and increase hepatic stress [26, 40]. A similar pattern was observed in the gastric tissue. Fish fed CK and the lower BSFL replacement diets (BSFL10–BSFL30) showed no marked stomach lesions, which agrees with previous reports that moderate insect meal inclusion does not necessarily induce gastric damage [41]. However, fish fed BSFL40 and BSFL50 showed inflammatory changes, including altered gland distribution, lymphocytic infiltration, eosinophilic granulocytes, and vascular dilation. These responses may also be associated with increased dietary chitin at higher replacement levels, which has been linked to gastrointestinal irritation and inflammation in fish [42].

Transcriptomic analysis provided further insights into the molecular responses to graded BSFL replacement. The strongest liver transcriptional response was observed in the BSFL30 group, suggesting that this treatment induced substantial metabolic adaptation before the more severe histological alterations seen at higher inclusion levels. Importantly, these transcriptomic responses should be interpreted together with the biochemical and histological findings rather than as isolated molecular signals. qRT‐PCR validation of representative DEGs showed expression trends broadly consistent with the RNA‐seq data, thereby supporting the reliability of the major transcriptomic patterns identified in the liver under graded BSFL replacement.

In the liver, the observed transcriptional changes were closely associated with dietary BSFL inclusion. Zarantoniello et al. [43] reported that increasing levels of full‐fat BSF meal over 6 months caused no significant adverse effects in zebrafish, as intestinal histology showed no inflammation and stress‐ or immune‐related markers remained unchanged. In the present study, several genes associated with adaptive responses to alternative feeds, including *aifm2*, *wee2*, *chek2*, *cdkn1d*, *mdm4*, *casp7*, *casp9*, *zgc:55558*, *siva1*, *anapcf13*, and *myca*, were upregulated. These genes are thought to participate in cell‐cycle checkpoint regulation and apoptosis‐related processes [44].

In particular, the BSFL30 group showed marked upregulation of apoptosis‐related genes, together with increased expression of genes associated with anti‐apoptotic regulation. This pattern suggests an active cellular response involving cell‐cycle control and DNA damage repair in the liver [45]. Overall, these transcriptional changes likely reflect the physiological and molecular adaptation of hybrid grouper to graded BSFL replacement. As the dietary replacement level increased, regulation of cell‐cycle progression and apoptosis appeared to become more prominent, possibly to maintain cellular homeostasis under nutritional stress. Similar studies have shown that the altered expression of these genes may reflect stress‐related and metabolic responses to alternative feed ingredients [46, 47].

In the muscle tissue, the altered expression of genes such as *CCND2A*, *IKBKG*, *GSK3B*, *TNFRSF1A*, *MAPK8*, and *IKBKB* in the BSFL30 group suggests an active physiological response to dietary BSFL inclusion. These genes are associated with cell‐cycle regulation, metabolic demand, stress signaling, apoptosis, and inflammatory or immune responses [46, 48–51]. Their coordinated regulation indicates that BSFL30 elicited substantial transcriptional adjustments in muscle, likely reflecting increased metabolic activity and cellular adaptation at this replacement level.

Genes related to the amino acid metabolism were also markedly affected by BSFL replacement. In the liver, the BSFL30 group showed upregulation of genes such as *arg2*, *gamt*, *p4ha2*, *p4ha1b*, *shmt2*, *kmt2a*, *kmt2bb*, *sms*, *ehmt1a*, *setd2*, and *plod2*, indicating active regulation of amino acid metabolism, methylation, polyamine synthesis, cellular repair, and structural protein synthesis [45, 52–54]. These responses suggest that moderate BSFL inclusion stimulated metabolic pathways involved in nutrient utilization and cellular maintenance.

A similar pattern was observed in muscle, where amino acid metabolism‐related genes were more highly expressed in the BSFL30 group than in the FM control group. Upregulation of genes such as arg1, prodh2, and agxtb suggests enhanced amino acid turnover and related metabolic regulation in response to BSFL inclusion. Some of these genes are also associated with histone methylation, amine metabolism, and signaling pathways, indicating broader effects on gene regulation and cellular responses to dietary change [38, 46, 55]. Together, these findings suggest that moderate BSFL replacement, particularly at 30%, induced coordinated transcriptional adjustments in the liver and muscle that may support metabolic adaptation, whereas higher inclusion levels could impose greater physiological burden.

This study revealed altered expression of lipid metabolism‐related genes in the liver of hybrid grouper‐fed diets with increasing levels of BSFL. In particular, the upregulation of genes such as *gpat2*, *hsd17b12a*, *ache*, *acaa2*, *gpcpd1*, *pla2g15*, and *pisd* in the BSFL30 group suggests enhanced lipid metabolic activity under moderate BSFL replacement [44]. In contrast, the downregulation of other lipid‐related genes at higher replacement levels may indicate the suppression or redistribution of specific lipid metabolic pathways in response to dietary stress [56].

The BSFL30 group also showed high expression of several other lipid metabolism‐related genes, including *acsl5*, *si:ch73-21k16.1*, *lclat1*, *acsbg2*, *plpp2b*, *ptgs2b*, *gpx7*, *lipg*, *dgkab*, and *pla2g4ab*, suggesting active regulation of lipid synthesis, remodeling, and transport at this replacement level [44]. Similarly, Vargas et al. [46] reported that altered expression of lipid metabolism‐related genes in grouper muscle was associated with changes in lipid utilization and energy conversion.

Several genes, including *lpin1*, *chkb*, *dgat1a*, *agpat9l*, *lipg*, *pld1a*, and *acsbg2*, showed decreased expression with increasing replacement of FM by BSFL. This pattern suggests that high BSFL inclusion may trigger adaptive regulation of lipid synthesis, degradation, and transport pathways, highlighting the complex effects of BSFL substitution on lipid metabolism in grouper. Further work is needed to clarify the molecular mechanisms and physiological consequences of moderate and high BSFL inclusion levels.

## 5. Conclusion

In conclusion, BSFL can be used as a partial substitute for FM in hybrid grouper diets, but its effects depend strongly on the dietary inclusion level. Among the tested diets, the best observed growth performance was obtained at 20% replacement, whereas quadratic regression based on BWG and SGR suggested lower mathematical optima of ~8%. Additional evaluations of serum biochemistry, digestive enzyme activity, histopathology, transcriptomics, and qRT‐PCR showed that replacement levels above 20% were associated with physiological stress, tissue alteration, and broader metabolic reprogramming, even though survival remained high. Therefore, the value of this study lies not only in estimating an optimal replacement level but also in defining a practical and biologically safer range for BSFL inclusion in hybrid grouper culture. The qRT‐PCR validation further supported the reliability of the major transcriptomic patterns identified in this study, particularly those related to apoptosis, amino acid metabolism, and lipid metabolism in the liver tissue.

## Author Contributions

Yan Chen, Minyi Zhong, and Jiun‐Yan Loh contributed to the study design. Yan Chen, Jiun‐Yan Loh, and Hai Huang contributed to data interpretation. Yan Chen, Minyi Zhong, Hai Huang, and Jiun‐Yan Loh assisted with sample processing, analysis, and experiments. Minyi Zhong, Bing Chen, Junming Cao, and Yan Chen contributed to data visualization, genomic analysis, and statistical analysis. Yan Chen drafted the manuscript. Jiun‐Yan Loh revised and improved the manuscript. Hai Huang and Jiun‐Yan Loh provided supervision.

## Funding

This study was supported by the Hainan Province Science and Technology Special Fund (Grant SQ2024XDNY0083), the Major Science and Technology Program of the Yazhou Bay Innovation Institute of Hainan Tropical Ocean University (Grant 2022CXYZD001), the National Natural Science Foundation of China (Grant 32160861), and the Major Science and Technology Plan of Hainan Province (Grant ZDKJ2021017). Special thanks are extended to the College of Fisheries and Life Science at Hainan Tropical Ocean University (Sanya, China), the Hainan Key Laboratory for Conservation and Utilization of Tropical Marine Fishery Resources, the Key Laboratory of Utilization and Conservation for Tropical Marine Bioresources of the Ministry of Education, and the Yazhou Bay Innovation Institute at Hainan Tropical Ocean University, Sanya 572022, China, for their assistance in this study.

## Disclosure

All authors have read and agreed to the published version of the manuscript.

## Ethics Statement

The authors adhered to all applicable international, national, and institutional guidelines for the care and use of animals. The use of animals was approved by the Institutional Animal Care and Use Committee of Hainan Tropical Ocean University.

## Consent

The authors have nothing to report.

## Conflicts of Interest

The authors declare no conflicts of interest.

## Data Availability

The data that support the findings of this study are available from the corresponding author upon reasonable request.
